# Integrative Multi-Omics Elucidates the Molecular Mechanisms Underlying Sweet-Mellow Taste Formation During Withering of Niangniang Tea (Large-Leaf Yellow Tea)

**DOI:** 10.3390/foods15162896

**Published:** 2026-08-19

**Authors:** Xia Lv, Yanxia Wang, Hao Guan, Li Lu, Juan Yang, Xiaosong Li, Hao Huang, Xiaozhen Huang, Litang Lu

**Affiliations:** 1 College of Tea Science, Guizhou University, Guiyang 550025, China; 2 Tea Research Institute, Chongqing Academy of Agricultural Sciences, Yongchuan, Chongqing 402160, China; 3 Tongren Academy of Agricultural Sciences, Tongren 554300, China; 4 Guizhou Poliu Tribute Tea Agricultural Development Co., Ltd., Zhenfeng 562200, China

**Keywords:** yellow tea, withering, flavor quality, metabolomics, transcriptomics

## Abstract

Withering is a critical but poorly understood step in yellow tea processing. This study integrated metabolomics (ultra-performance liquid chromatography–tandem mass spectrometry, UPLC-MS/MS), transcriptomics (RNA sequencing, RNA-seq), and sensory evaluation to investigate dynamic changes in key metabolites and gene expression during withering (0–10 h) of Niangniang tea (NNT). Transcriptomic analysis was performed on leaves sampled directly during withering, while metabolomic profiling and sensory evaluation were conducted on finished teas processed from the corresponding withered leaves. A total of 1064 metabolites were identified. Sensory evaluation showed that 8 h of withering yielded the highest sweet-mellow taste scores. Theanine, L-aspartic acid, and L-arginine peaked at 8 h, while galloylated catechins decreased. Key regulatory genes (*CsTA*, *CsSCPL*, *CsPDX2.1*) were identified, with expression patterns correlating with sweet-related amino acid accumulation and catechin conversion. Integrative analysis delineated a molecular roadmap in which downregulation of *CsPDX2.1* after 6 h was associated with reduced theanine hydrolysis, and upregulation of *CsTA* correlated with galloylated catechin conversion, collectively contributing to sweet-mellow taste. These findings provide a scientific basis for optimizing withering duration in large-leaf yellow tea production.

## 1. Introduction

Tea is one of the most widely consumed beverages globally, valued for its complex sensory characteristics and diverse health-promoting metabolites [1,2]. Among the various tea categories, yellow tea holds a unique position by its “yellow leaves and yellow liquor” appearance [3], bioactive compounds with antioxidant, anticancer, antimicrobial, and gastrointestinal effects [4], and a mellow flavor that has attracted increasing international interest [5]. The flavor profile of tea is predominantly determined by post-harvest processing [6,7,8]. Most yellow-tea research has focused on the yellowing stage [9,10,11,12], while less attention has been paid to withering, the initial and critical step involving controlled dehydration. Moderate withering reduces astringent catechins and enhances sweet-tasting compounds such as theanine and soluble sugars [3]. However, excessive withering may compromise the characteristic freshness of yellow tea. Despite its importance, systematic studies on withering in yellow tea remain scarce, with most existing research focused on green, oolong, or black teas.

In black and oolong teas, prolonged withering attenuates bitterness by facilitating catechin oxidation and polymerization [13]. Yellow tea, by contrast, undergoes only mild oxidation, implying that its flavor modulation relies on a distinct suite of enzymatic pathways. High-throughput omics, particularly metabolomics and transcriptomics, now enable real-time mapping of metabolite trajectories and their genetic regulators [14]. Key enzymes such as tannase (*CsTA*) and serine carboxypeptidase-like acyltransferases (*CsSCPL*) drive the interconversion of astringent galloylated catechins into their milder non-galloylated counterparts [15,16], while theanine hydrolysis, central to umami perception, is similarly governed by amino-acid-metabolizing enzymes activated during processing [17].

In yellow tea, the withering process is adjusted according to leaf maturity, cultivar, and environmental conditions [11,18,19]. For example, tender buds typically require shorter withering durations to avoid excessive moisture loss, while more mature leaves may undergo extended withering to promote enzymatic activity. Niangniang tea (NNT), a rare large-leaf yellow tea variety originating from Guizhou Province, China, is traditionally produced from the leaves of ancient tea trees (*Camellia sinensis* var. assamica) that grow in high-altitude regions and yield leaves 3–4 inches in length. These wild ancient tea trees contain elevated levels of secondary metabolites, attributed to their long growth cycles and adaptation to natural stressors [20]. These characteristics, together with unique terroir attributes and traditional processing techniques, contribute to a complex flavor profile that distinguishes NNT from conventional yellow teas. While the second bunch-drying stage has been shown to enhance its characteristic sweet flavor [21], standardized protocols for the withering process remain lacking. In traditional NNT production, withering durations are determined empirically, ranging arbitrarily from 2 to 12 h. This variability leads to inconsistent flavor profiles and limits the tea’s market potential.

This study integrated UPLC–MS/MS-based metabolomics, RNA-seq transcriptomics, and sensory evaluation to elucidate the molecular mechanisms underlying sweet-mellow taste formation during withering of Niangniang tea (NNT). We hypothesized that sweet-mellow taste formation involves coordinated changes in theanine and catechin metabolism, with potential regulatory roles of *CsTA*, *CsSCPL*, and *CsPDX2.1* (pyridoxal phosphate-dependent decarboxylase). Our objectives were to profile dynamic metabolite, gene trajectories, construct regulatory networks, and determine the optimal withering duration. This study provides a scientific framework for withering optimization in NNT and serves as a reference for precision processing in specialty teas.

## 2. Materials and Methods

### 2.1. Preparation of Tea Samples

In this study, fresh tea leaves (one bud and three leaves) of the local ancient tea tree population (*Camellia sinensis* var. assamica) were collected from Poliu Village, Zhenfeng City, Guizhou Province (N 23°26′41″, E 104°32′55″, 1053.08 m), China, on 4 July 2025. Approximately 2.5 kg of fresh leaves per replicate were evenly spread (2–3 cm thickness) on bamboo sieves and subjected to withering under controlled conditions (23–26 °C, 55–65% relative humidity). Samples were collected at 0, 2, 4, 6, 8, and 10 h during withering, with untreated leaves (0 h) serving as the control. Each sampling time point included three independent biological replicates. After withering, one portion of each sample was immediately frozen in liquid nitrogen and stored at −80 °C for transcriptomic analysis, as this approach captures the transient gene expression dynamics during withering before RNA is degraded by subsequent processing steps. The remaining leaves were processed into finished tea following standardized procedures for metabolomic and sensory evaluation, since the final flavor phenotype reflects the integrated outcome of the entire manufacturing chain:

Fixation: Using a roller fixation machine at 320–350 °C for 3–4 min.

Rolling: At room temperature under light pressure for 30–40 min.

Yellowing: At 25–28 °C and 65–70% relative humidity for 24 h.

Shaping: Hand rolling for 15–20 min and shaping.

Slow drying: At 50–60 °C for 4–5 h, and sun-drying at 28–30 °C for 7 days, until the moisture content reached below 6%.

The final tea samples were labeled as CK (0 h), Ya (2 h), Yb (4 h), Yc (6 h), Yd (8 h), and Ye (10 h), yielding a total of 18 samples (six time points × three biological replicates).

### 2.2. Sensory Evaluation

Sensory evaluation was conducted following the Chinese national standard GB/T 23776-2018 [22]. A panel of ten certified tea evaluators (each with >5 years of experience) evaluated the tea samples after standardized training and calibration. For each sample, 3 g of tea was infused with 150 mL of boiling water (100 °C) for 5 min. The infusion was filtered and assessed for appearance, liquor color, aroma, and taste using a weighted scoring system. Total sensory scores were calculated accordingly.

### 2.3. Metabolomic Analysis

Freeze-dried tea powder was prepared from the final processed samples (CK, Ya, Yb, Yc, Yd, Ye) using a laboratory freeze-dryer (−50 °C, <10 Pa, 48 h). The dried material was ground to a fine powder and passed through a 60-mesh sieve. Metabolite extraction was performed as described by Shen et al. [23] with modifications: 2.0 g of powder was mixed with 40 mL of 70% methanol (*v*/*v*), ultrasonicated for 30 min at 25 °C, and incubated for 4 h. The residue was re-extracted with an additional 40 mL of 70% methanol under the same conditions. The combined supernatants were centrifuged at 10,000× *g* for 10 min, diluted 40-fold with methanol to reduce matrix effects and bring metabolite concentrations within the instrument’s linear detection range, and filtered through a 0.22 μm membrane. Quality control (QC) samples were prepared by pooling equal volumes of all extracts. Untargeted metabolomic profiling was performed using a triple quadrupole LC–MS system (TSQ Fortis™ Plus, Thermo Fisher Scientific, USA) with an ESI Turbo IonSpray interface operating in positive and negative ion modes. System control and data acquisition were conducted using Analyst 1.6.3 software. Chromatographic separation and ionization conditions followed previously described protocols [24,25]. Raw MS data were processed using Analyst (v1.6.3) and Multiquanta software [26].

### 2.4. Transcriptome Analysis

Total RNA was extracted using the TaKaRa MiniBEST Plant RNA Extraction Kit (TaKaRa, Kyoto, Japan) following the manufacturer’s protocol. RNA integrity and concentration were assessed via 1% agarose gel electrophoresis and a NanoDrop ND-1000 spectrophotometer (Thermo Fisher Scientific, Waltham, MA, USA); samples with RNA integrity number (RIN) ≥ 7.0 were used for library construction. cDNA libraries were constructed using the NEBNext^®^ Ultra™ RNA Library Prep Kit for Illumina (NEB, Ipswich, MA, USA) following the manufacturer’s instructions, and sequenced on the Illumina NovaSeq 6000 platform (paired-end 150 bp, PE150) by Biomarker Technology Co., Ltd. (Beijing, China). Eighteen libraries (six time points × three biological replicates) were sequenced. Raw sequencing reads in FASTQ format were subjected to quality control using FastQC (v0.11.9) and Trimmomatic (v0.39) to remove adapter sequences and low-quality bases (Q < 20). Clean reads were aligned to the Camellia sinensis reference genome (CSS genome assembly; Tea Plant Information Archive, http://tpia.teaplant.org) using HISAT2 (v2.2.1) with default parameters. Aligned reads were sorted and indexed using SAMtools (v1.10), and gene-level read counts were quantified using featureCounts (v2.0.1) with the Ensembl plant gene annotation (GFF3). Differential expression analysis was performed using the DESeq2 R package (v1.36.0), and genes with |log_2_ fold change (FC)| ≥ 1 and adjusted *p*-value (false discovery rate, FDR) < 0.05 were considered differentially expressed genes (DEGs). The RNA-seq data analysis pipeline followed the workflow described by Qiao et al. [27]. Raw sequencing data have been deposited in the NCBI Sequence Read Archive under accession number PRJNA869358.

Weighted gene co-expression network analysis (WGCNA) was performed using the WGCNA R package to identify gene modules associated with flavor-related metabolites. Briefly, after filtering genes with mean expression > 1 and coefficient of variation > 0.1 across all samples, the remaining 19,527 genes were used for network construction. To ensure a scale-free topology, the soft-thresholding power (β) was determined using the pickSoftThreshold function, with powers ranging from 1 to 20 and the criterion of scale-free topology fit index (R^2^) > 0.85. A β value of 14 was selected for subsequent analysis. The topological overlap matrix (TOM) was computed, and genes were clustered via hierarchical clustering using the dynamic tree-cut algorithm (cutreeDynamic) with a minimum module size of 30 and a module merging threshold of 0.25. Modules with highly similar expression profiles were merged using the mergeCloseModules function with a cut height of 0.25. The module eigengene (ME) for each module was calculated as the first principal component of its expression matrix. Pearson’s correlation coefficients between MEs and 12 characteristic metabolites (including L-glutamic acid, L-phenylalanine, L-aspartic acid, L-arginine, threonine, CG, GCG, ECG, EGCG, C, EC, and EGC) were calculated to identify trait-associated modules. Modules with |correlation| > 0.6 and *p* < 0.05 were considered significantly associated with the corresponding metabolites. Network visualization was performed using Cytoscape 3.9.1. Correlation between qRT-PCR and RNA-seq data was evaluated using Pearson’s correlation coefficient.

### 2.5. Data Analysis

Statistical analyses were performed using SPSS version 25.0 (SPSS Inc., Chicago, IL, USA). One-way analysis of variance (ANOVA) followed by Tukey’s post hoc test was used to evaluate significant differences among groups (*p* < 0.05). Figures were generated using Origin 2023 (OriginLab Co., Northampton, MA, USA). Principal component analysis (PCA) and orthogonal partial least squares discriminant analysis (OPLS-DA) were conducted using SIMCA-P version 14.0. The quality and predictive ability of each OPLS-DA model were evaluated using the cumulative parameters R^2^X(cum), R^2^Y(cum), and Q^2^(cum). Model validity was further assessed by CV-ANOVA (analysis of variance testing of cross-validated predictive residuals), with *p* < 0.05 indicating a valid model, and by permutation testing (200 random permutations) to exclude overfitting. Metabolites with variable importance in projection (VIP) > 1 were considered important for group discrimination Kyoto Encyclopedia of Genes and Genomes (KEGG) enrichment analysis and heatmap visualization were performed using TBtools (v1.082) [28].

## 3. Results and Discussion

### 3.1. Sensory Changes During Withering

**Figure 1:**
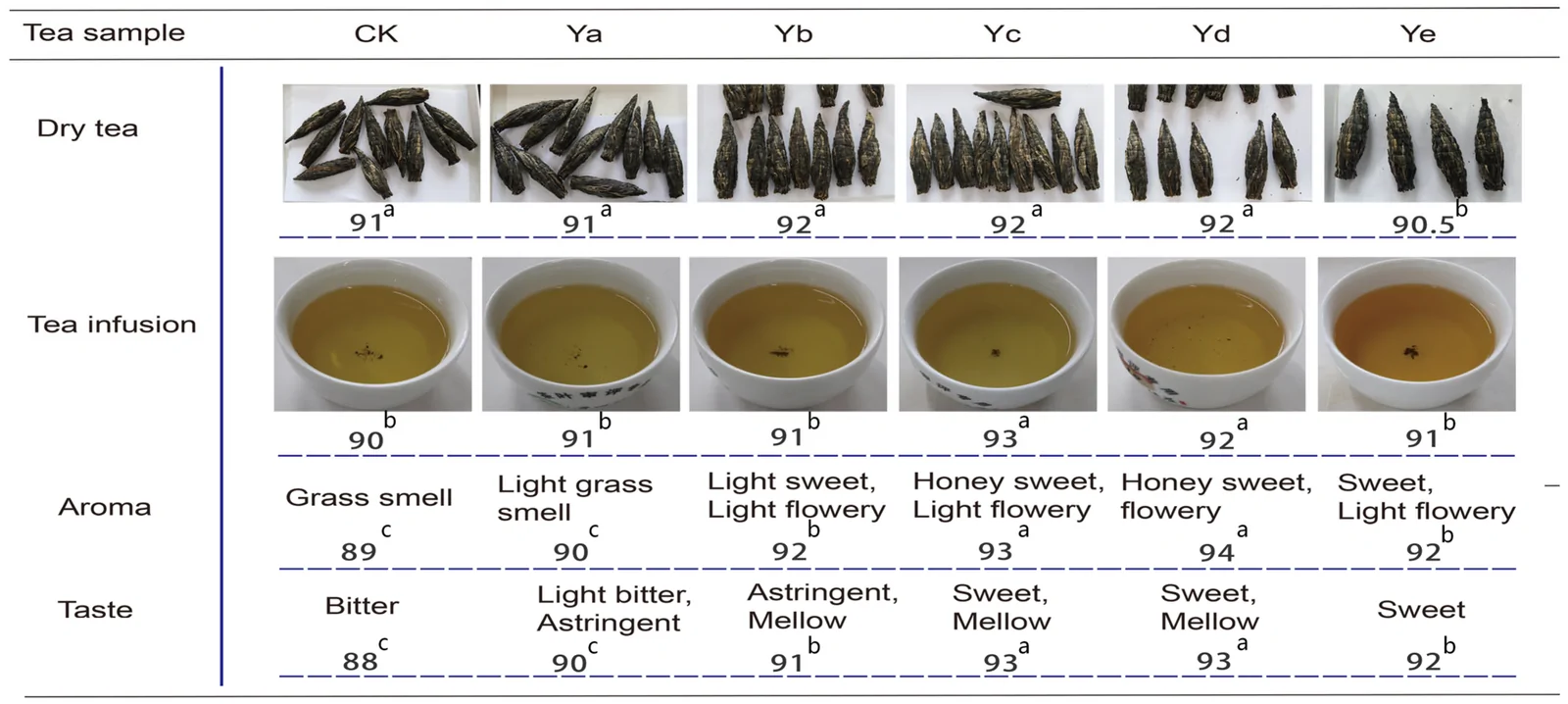
Sensory evaluation of NNT samples subjected to different withering durations. (including the appearance and liquor image, aroma and taste description and score). Different lowercase letters indicate statistically significant differences (*p* < 0.05).

Withering is a key process affecting the flavor of NNT. The sensory properties of NNT samples subjected to different withering durations were evaluated (Figure 1). No significant differences were observed in the appearance of the dried tea; however, liquor color, aroma, and taste varied considerably, particularly the latter two attributes. With increasing withering time, NNT developed a pronounced sweet flavor, reflected in both aroma and taste. Among all treatments, Yd (8 h) received the highest sensory score, significantly higher than CK, Ya, Yb, and Ye (one-way ANOVA, *p* < 0.05, Tukey’s post hoc test), while Yc (6 h) and Yd (8 h) obtained comparable scores with no significant difference between them (*p* > 0.05). These results indicate that withering duration significantly influences the flavor quality of NNT, with an optimal range of 6–8 h.

### 3.2. Dynamic Changes in Key Metabolites of NNT

**Figure 2:**
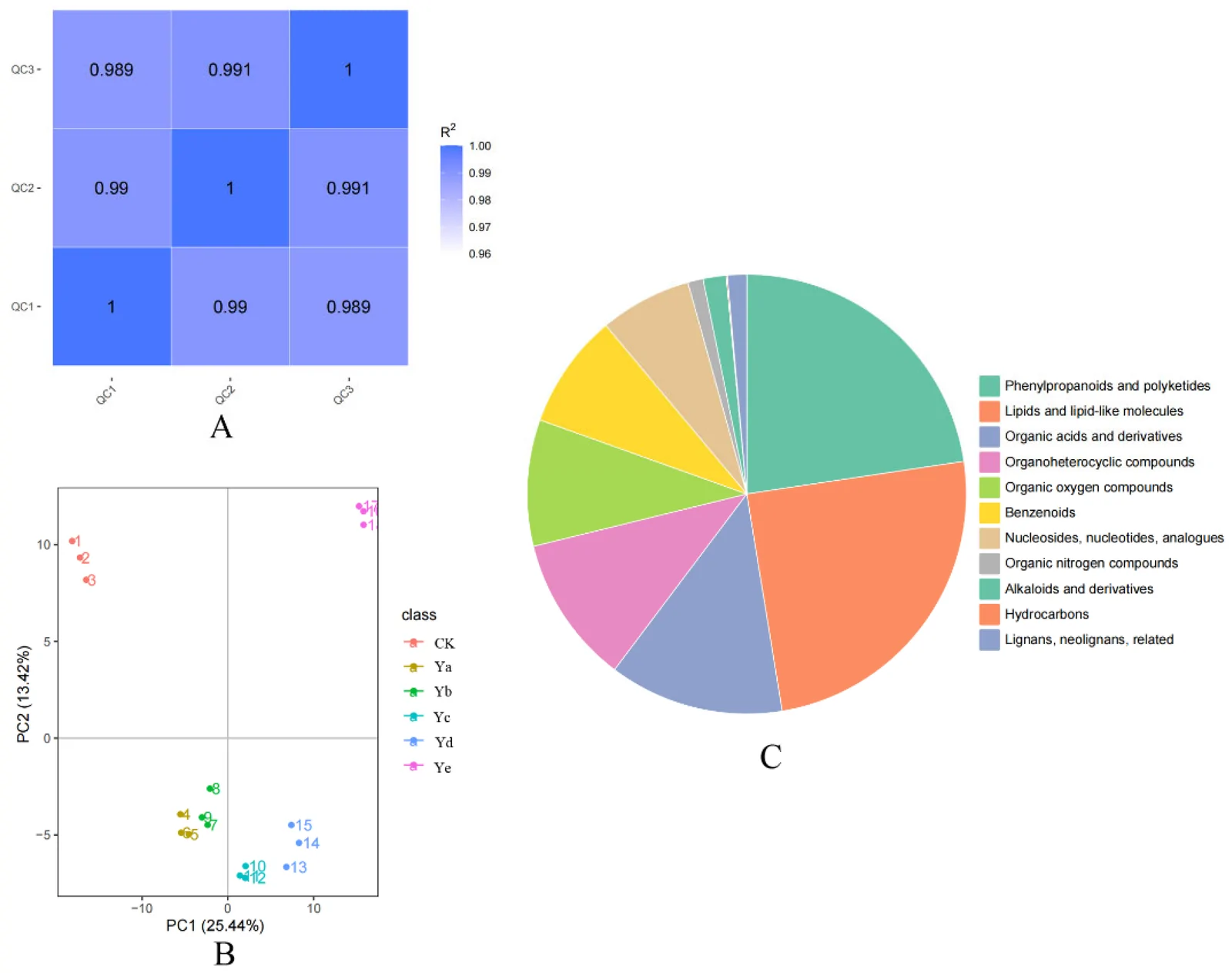
Quality control and metabolomic profiling of NNT during withering. (**A**) Pearson correlation heatmap of QC samples (R^2^ > 0.95), confirming analytical stability. (**B**) Principal component analysis of the identified differential metabolites. (**C**) Classification of the 1064 identified metabolites of NNT during withering.

Untargeted UPLC–MS/MS analysis was performed for CK and five NNT samples at five different withering stages in positive and negative ion modes. The high Pearson correlation (R^2^ > 0.95) indicated that the entire process was stable and reliable (Figure 2A). QC samples were added to the test to evaluate the metabolomic performance. PCA was performed on the samples to determine overall metabolite differences between groups and changes within groups. The score plot showed that the QC samples were clustered in the center, and the three biological replicates at each time point clustered closely, indicating that the metabolomic analysis was repeatable and reliable (Figure 2B). The samples from different withering periods were completely separated in the PCA diagram, indicating that the process induced a significant change in the metabolite profile.

A total of 1064 metabolites were detected from 18 NNT samples, including 264 lipids and lipid-like molecules, 241 phenylpropanoids and polyketides, 137 organic acids and derivatives, 90 benzenoids, 99 organic oxygen compounds, and 116 organ heterocyclic compounds. Of these, the three major categories that significantly affected the tea flavor were phenylpropanoids, lipids, and organic acids, accounting for approximately 60% of the total (Figure 2C).

#### 3.2.1. Identification of Differentially Accumulated Metabolites (DAMs)

**Figure 3:**
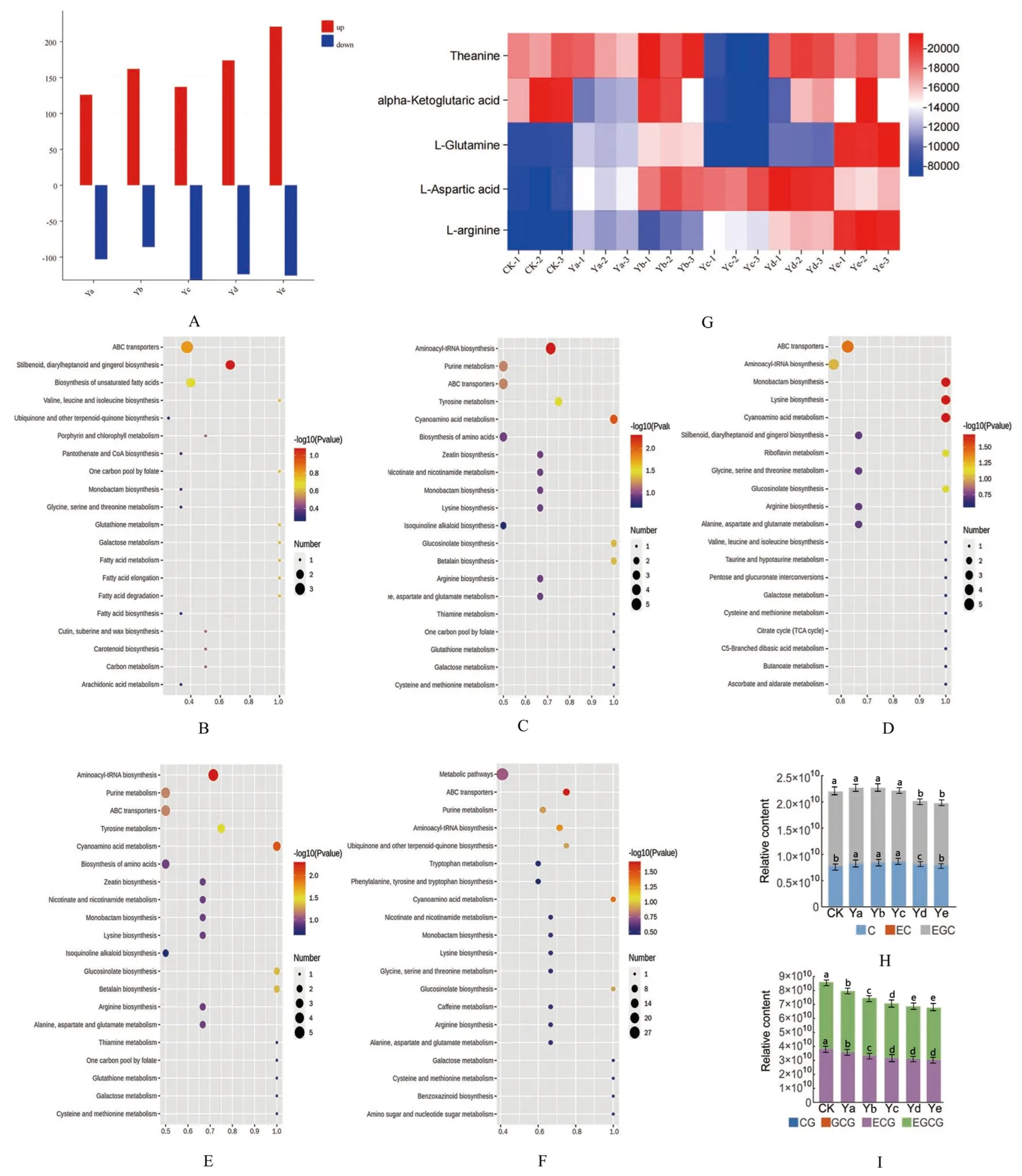
Differential metabolite accumulation and KEGG pathway enrichment during NNT withering. (**A**) Changes in the number of differential metabolites with the increase in withering time. (**B**) KEGG enrichment of differential metabolites between Ya and CK. (**C**) KEGG enrichment of differential metabolites between Yb and CK. (**D**) KEGG enrichment of differential metabolites between Yc and CK. (**E**) KEGG enrichment of differential metabolites between Yd and CK. (**F**) KEGG enrichment of differential metabolites between Ye and CK. (**G**) Changes in amino acids primarily regulated in the metabolic pathways of aminoacyl-tRNA biosynthesis (map00970), arginine biosynthesis (map00220), and ABC transporters (map02010) during the withering process. (**H**) Changes in the content of galloylated catechins during the withering process. (**I**) Changes in the content of non-galloylated catechins during the withering process. Ya, Yb, Yc, Yd, Ye and CK represent 2 h, 4 h, 6 h, 8 h, 10 h and control check (fresh leaves). Different lowercase letters indicate statistically significant differences (*p* < 0.05).

To investigate the changes in taste-related metabolites during withering, the metabolite profiles at each time point were compared with those of fresh leaves (CK), generating five paired comparison groups. The OPLS-DA models for all five comparison groups were validated by CV-ANOVA (*p* < 0.05) and permutation tests (200 permutations), with R^2^Y(cum) and Q^2^(cum) values indicating good fit and predictive ability. Metabolites with VIP > 1 and |log_2_FC| ≥ 1.8 were considered differentially accumulated metabolites (DAMs) (Table S1). As shown in Figure 3A, the number of DAMs increased with prolonged withering, and up-regulated DAMs outnumbered down-regulated ones in all comparison groups. These findings indicate active metabolite formation and transformation during NNT withering.

#### 3.2.2. KEGG Enrichment Analysis and Dynamic Changes of Key Taste-Related Metabolites

To elucidate the dynamic changes in metabolic pathways during the withering process of NNT, KEGG enrichment analysis was performed on the differential metabolites identified across the five comparison groups (Ya-Ye vs. CK). The top 20 enriched pathways for each comparison group are presented in Figure 3B–F, with significant enrichment defined as *p* ≤ 0.05. The results revealed a clear temporal progression in pathway activation. In the early withering stage (Ya, 2 h), the most significantly enriched pathways were concentrated in primary metabolism, including biosynthesis of amino acids (map01230) and phenylalanine metabolism (map00360), with relatively low enrichment ratios (Figure 3B), indicating the initiation of metabolic responses to dehydration stress. From 4 h (Yb) to 8 h (Yd), the number and significance of enriched pathways increased markedly. In Yb (4 h), flavonoid biosynthesis (map00941; Ratio = 0.35) and phenylalanine metabolism (map00360; Ratio = 0.40) emerged as key pathways (Figure 3C), suggesting the onset of secondary metabolism. By Yc (6 h) and Yd (8 h), pathways related to amino acid metabolism and secondary metabolite biosynthesis became highly enriched (Figure 3D,E). Notably, at Yd (8 h), the enrichment ratio of biosynthesis of amino acids (map01230) reached 0.58, and tryptophan metabolism (map00380) exhibited a Ratio of 0.62, reflecting a peak in metabolic activity associated with sweet and umami compound accumulation. In contrast, at Ye (10 h), the enrichment of several pathways declined, with some showing negative enrichment values (Figure 3F), such as lysine degradation (map00310) and carotenoid biosynthesis (map00906), suggesting a shift toward catabolic processes and possible metabolic attenuation after prolonged withering. Across all comparison groups, pathways consistently enriched and statistically significant (*p* ≤ 0.05) included phenylalanine, tyrosine and tryptophan biosynthesis (map00400), aminoacyl-tRNA biosynthesis (map00970), arginine biosynthesis (map00220), and ABC transporters (map02010). These pathways were particularly evident in Yb, Yc, and Yd, highlighting their central role in regulating key taste-active compounds, including amino acids and catechins, during the formation of the sweet-mellow flavor in NNT.

Building on the KEGG enrichment results, the dynamic changes of representative metabolites within these key pathways were further examined. As shown in Figure 3G–I, the contents of L-aspartic acid and L-arginine increased continuously with prolonged withering time, particularly L-aspartic acid, which peaked in Yd (8 h). In contrast, L-glutamine and alpha-ketoglutaric acid showed fluctuating trends, reaching their lowest levels at Yc (6 h) before gradually increasing after 8 h. Theanine content declined slowly initially, reaching its lowest point in Yc (6 h), then surged rapidly to its highest level by 8 h, and stabilized thereafter. This pattern suggests a potential metabolic coordination during withering: the decline in theanine at early stages (Ya–Yc) coincided with increased levels of L-glutamine and α-ketoglutaric acid, while the subsequent accumulation of theanine, L-aspartic acid, and L-arginine at Yd (8 h) aligned with the upregulation of their biosynthetic pathways (map01230, map00220). Although isotope tracing or flux analysis would be required to definitively establish precursor-product relationships, the observed temporal correlations are consistent with the known roles of glutamate as a key hub in theanine and amino acid metabolism. By 6 h, the accumulated glutamate may have supported enhanced production of theanine, L-aspartic acid, and L-arginine, culminating in the highest theanine content at Yd (8 h), with stabilization observed by 10 h. Thus, the optimal sensory quality (highest overall score) in Yd (8 h) could possibly be attributed to the synergistic accumulation of theanine, L-aspartic acid, and L-arginine, which collectively contribute to the tea’s sweet and mellow taste [4,29,30].

Furthermore, the metabolic pathways of phenylalanine/tyrosine metabolism (map00400, map00350), glyoxylate metabolism (map00630), and ABC transporters (map02010) collectively regulated catechin biosynthesis and conferred moderate bitterness and astringency via an integrated metabolic network. As withering progressed, the content of galloylated catechins decreased gradually, with a significant reduction at Yd (8 h) compared with CK (*p* < 0.05). Non-galloylated catechins, including C, GC, EC, and EGC, with simpler molecular structures, have less bitter taste. During the withering process, the content of non-galloylated catechins first increased and then decreased, with a peak at Yb (4 h), where the increase from CK to Yb was statistically significant (*p* < 0.05); EGC accounted for the largest proportion at this time point. The content of C reached its highest at Yd (8 h), whereas EC demonstrated a downward trend throughout withering. These changes are consistent with the activation of hydrolytic enzymes during withering; specifically, the decrease in galloylated catechins and the corresponding increase in gallic acid suggest the involvement of tannase (*CsTA*), which hydrolyzes the galloyl ester bonds of EGCG and ECG to release free gallic acid and generate non-galloylated catechins (EGC and EC) [15,16]. These results were in accordance with the observed reduction in bitter taste and the persistence of astringency in sensory evaluation (Figure 1). However, the metabolite composition underlying sweet taste and the potential mechanisms regulating sweet flavor formation during NNT withering remained to be investigated. Therefore, we performed metabolomic and transcriptomic analyses to explore these potential associations.

The temporal enrichment patterns of these pathways provide mechanistic insights into the flavor transition during NNT withering. At 6–8 h (Yc–Yd), the concurrent enrichment of amino acid biosynthesis (map01230) and arginine biosynthesis (map00220) appears to reflect an active metabolic reprogramming associated with the accumulation of sweet/umami-related amino acids. Specifically, the upregulation of these pathways may provide precursors such as glutamate and arginine, which are directly utilized for theanine and L-aspartic acid synthesis [3,31]. The sustained enrichment of aminoacyl-tRNA biosynthesis (map00970) may ensure the activation and availability of amino acids for protein turnover and secondary metabolite production, suggesting that the cell maintains active translation machinery to support stress-responsive metabolism during dehydration [7]. Meanwhile, the enrichment of ABC transporters (map02010) may suggest enhanced transmembrane transport of amino acids and secondary metabolites, potentially facilitating the intracellular redistribution and accumulation of taste-active compounds [3].

The peak enrichment at 8 h (Yd) may reflect a physiological “critical point” during withering: by this time, the leaf has lost sufficient moisture to trigger a full stress response, yet the cellular integrity and enzymatic activities remain sufficiently intact to support active biosynthesis rather than degradation. In contrast, at 10 h (Ye), the declining enrichment of these pathways, coupled with the appearance of negative enrichment values for catabolic pathways such as lysine degradation (map00310), appears to indicate a shift toward metabolic attenuation and catabolism, which may compromise flavor quality. This temporal trajectory aligns with the sensory data, where Yd (8 h) achieved the highest scores.

Mechanistically, the coordinated pathway activation at 8 h appears to enhance sweet-mellow perception through two complementary biochemical routes. First, the accumulation of theanine, L-aspartic acid, and L-arginine, all products of the enriched amino acid pathways, is known to contribute to the sweet and umami taste profiles of tea infusions [3,29,30], as these amino acids exhibit taste-active properties at concentrations present in processed tea. Second, the concurrent reduction in galloylated catechins, mediated by pathways including flavonoid biosynthesis (map00941), is associated with diminished bitterness and astringency [3,13], as these compounds are primary contributors to aversive taste sensations in tea. Collectively, the enhancement of sweet/umami-related metabolites, coupled with the attenuation of bitter/astringent compounds, likely underlies the superior sensory quality observed at 8 h.

### 3.3. Transcriptome Profiles and GSVA Analysis

**Figure 4:**
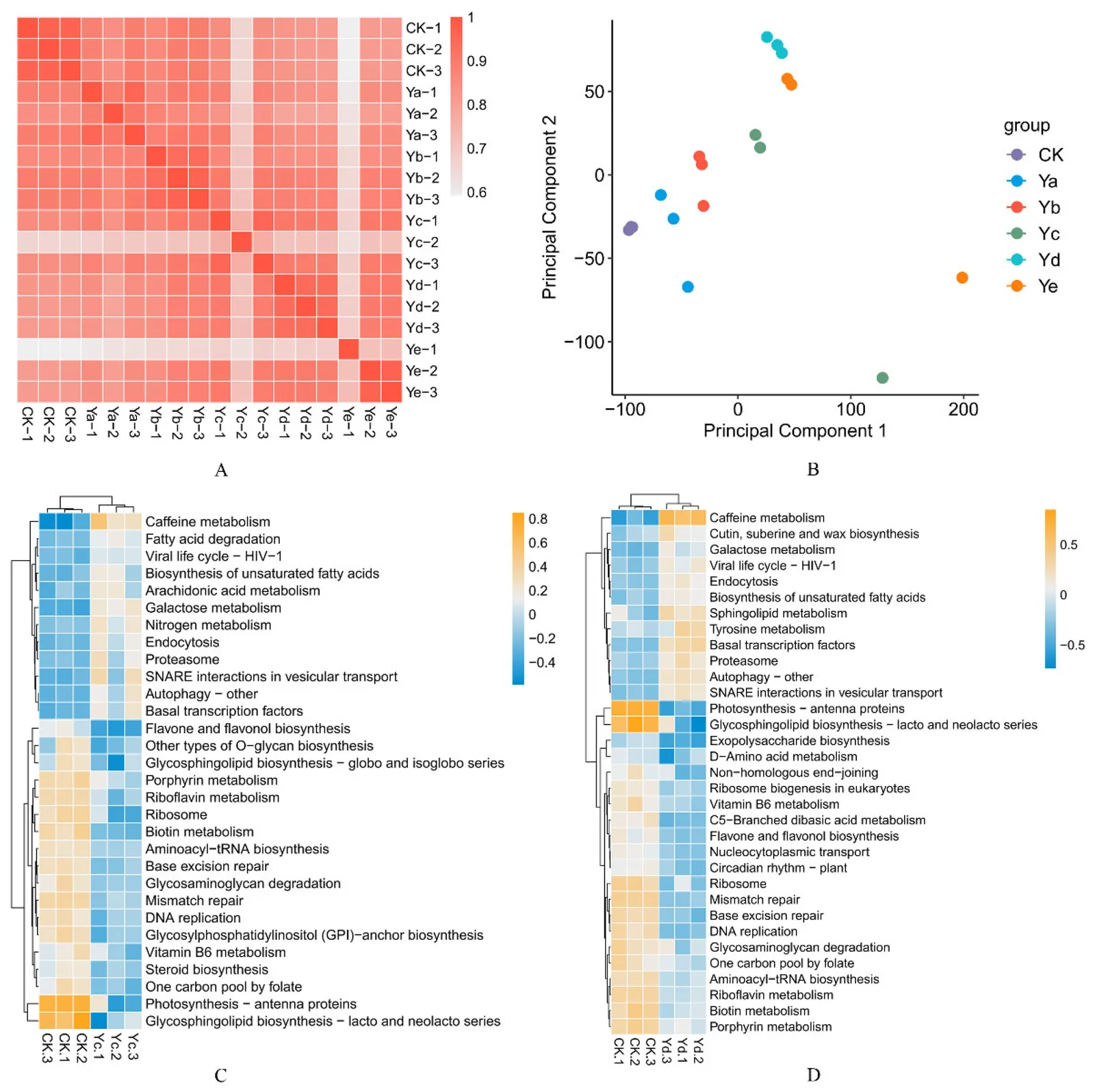
Transcriptome profiles and GSVA analysis during NNT withering. (**A**) Pearson correlation heatmap (R^2^ > 0.90), confirming reproducibility. (**B**) PCA plot showing clear separation of samples by withering time. (**C**,**D**) GSVA scores for selected metabolic pathways at Yc (6 h) and Yd (8 h); red indicates upregulated activity, blue indicates downregulated activity. At Yd (8 h), pathways related to amino acid and flavonoid biosynthesis showed enhanced activity, coinciding with sweet-related metabolite accumulation.

A total of 18 cDNA libraries (three biological replicates for each of the six NNT samples) were generated and sequenced for transcriptome profile analysis. We checked for correlations in gene expression (based on fragments per kilobase of transcript per million mapped reads (FPKM) values) among samples using the Pearson correlation coefficient (Figure 4A). The three biological replicates within each group exhibited high correlation (R^2^ > 0.90), indicating good reproducibility of the sequencing results. PCA analysis (Figure 4B) demonstrated that the six treatments could be well separated, with the six groups clustering into six distinct clusters according to the processing progression. This pattern was consistent with the clustering in the correlation heatmap, further confirming the reproducibility and reliability of the sequencing results (Figure 4C).

Gene set variation analysis (GSVA) was performed using the GSVA R package to evaluate pathway activity variations across different withering time points. The analysis was conducted on log_2_-transformed FPKM values using the default Gaussian kernel estimation of the cumulative density function, with the kcdf parameter set to “Gaussian”. A custom gene set collection was compiled from the KEGG pathways that were significantly enriched in the metabolomic analysis (as identified in Section 3.2.2), including amino acid biosynthesis (map01230), arginine biosynthesis (map00220), aminoacyl-tRNA biosynthesis (map00970), ABC transporters (map02010), and flavonoid biosynthesis (map00941). For each sample, GSVA generated a pathway-level enrichment score, where positive scores indicate relative upregulation and negative scores indicate relative downregulation of the pathway compared to the average across all samples. The resulting GSVA scores were used to compare pathway activity patterns between the optimal withering stages (Yc and Yd) and other time points. In the Yc group, down-regulation of nitrogen metabolism may reduce glutamate supply, where as stable expression of aminoacyl-tRNA biosynthesis may support activation of amino acids required for theanine synthesis. ABC transporters may be involved in theanine accumulation. The stable expression of flavone and flavonol biosynthesis pathways suggests that catechin synthesis is not significantly suppressed, though catechins may undergo oxidation to theaflavins and thearubigins, which are associated with improved sensory profiles in processed teas.

In the Yd group, enhanced tyrosine metabolism may provide precursors for theanine biosynthesis, as tyrosine can be transaminated to p-hydroxyphenylpyruvate [3]. Stable expression of aminoacyl-tRNA biosynthesis may support the activation of amino acids required for theanine synthesis. Notably, D-amino acid metabolism may modulate taste characteristics by influencing the stereoisomeric balance of L-theanine [2]. Activated flavone and flavonol biosynthesis pathways may be associated with catechin accumulation, while stable galactose metabolism may provide glycosyl donors for catechin glycosylation, potentially reducing astringency [32]. Additionally, alterations in sphingolipid metabolism may regulate transmembrane transport and accumulation of catechins by modulating membrane permeability [33].

### 3.4. Integrated Analysis of Metabolomics and Transcriptomics

**Figure 5:**
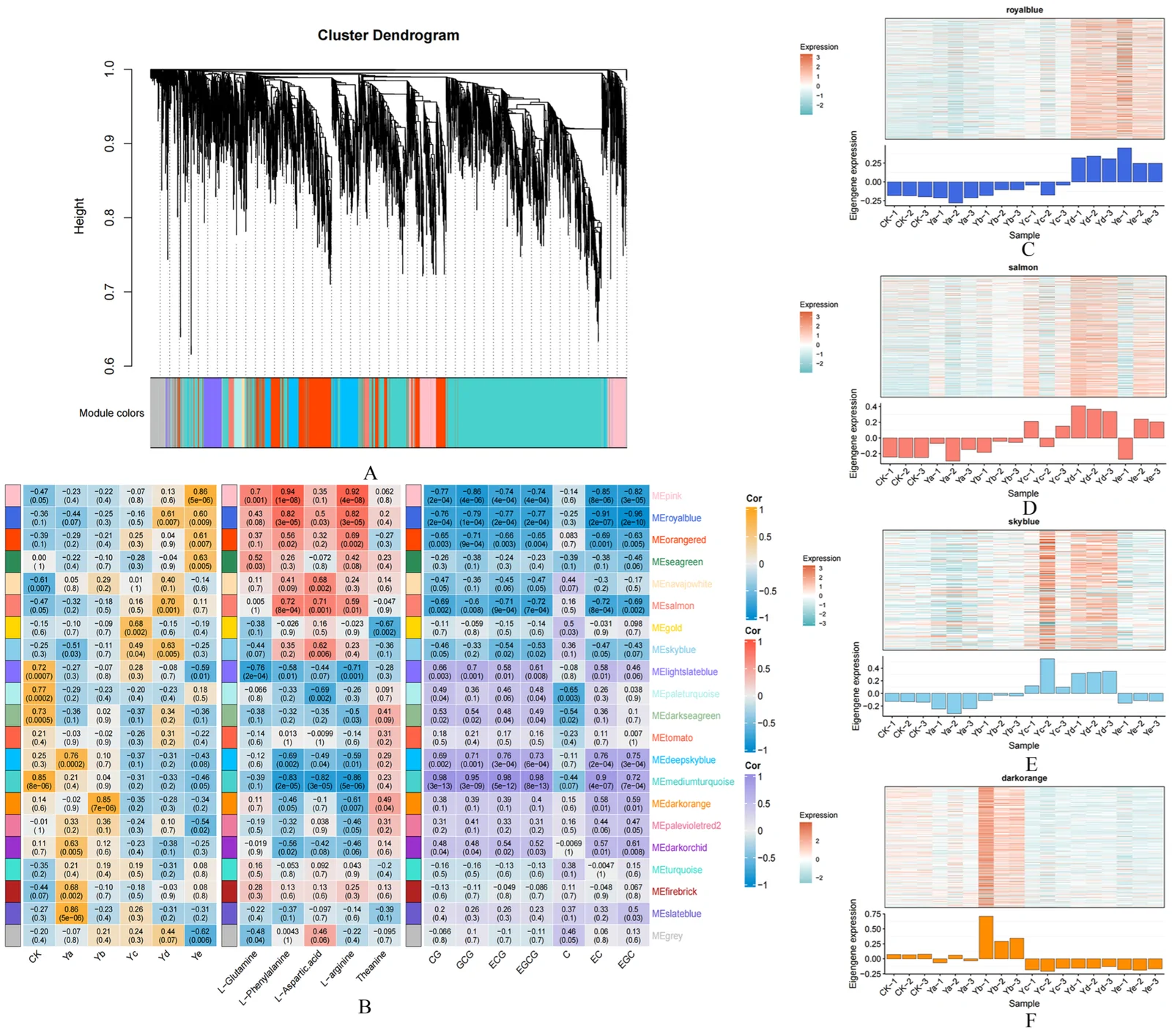
WGCNA of NNT during withering. (**A**) Hierarchical clustering dendrogram of 19,527 genes, with 21 co-expression modules identified. (**B**) Heatmap of correlations between module eigengenes and 12 taste-related metabolites; the royalblue, salmon, skyblue, and darkorange modules showed the strongest correlations (|r| > 0.6, *p* < 0.05) with withering time and key metabolites. (**C**–**F**) Expression heatmaps of genes within these four modules; red: high expression, blue: low expression.

Weighted gene co-expression network analysis (WGCNA) was performed to identify gene modules associated with the formation of sweet and mellow related compounds during NNT withering. After filtering (mean expression > 1, coefficient of variation > 0.1), 19,527 genes were retained, and 21 co-expression modules were identified (Figure 5A). Correlation analysis was performed between module eigengenes, sample groups, and the 12 characteristic metabolites identified as key taste-active compounds in the metabolomic analysis (Section 3.2.2), including L-glutamic acid, L-phenylalanine, L-aspartic acid, L-arginine, threonine, CG, GCG, ECG, EGCG, C, EC, and EGC. The four modules royalblue, salmon, skyblue, and darkorange showed strong correlations with both the withering time series and key taste-related metabolites (|correlation| > 0.6, *p* < 0.05; Figure 5B). Expression heatmaps of genes within these modules (Figure 5C–F) further confirmed their association with the accumulation of sweet and mellow related compounds. These modules likely contain key regulatory genes governing flavor formation during NNT withering.

### 3.5. Analysis of Sweet and Mellow Related Compound Metabolic Pathways

**Figure 6:**
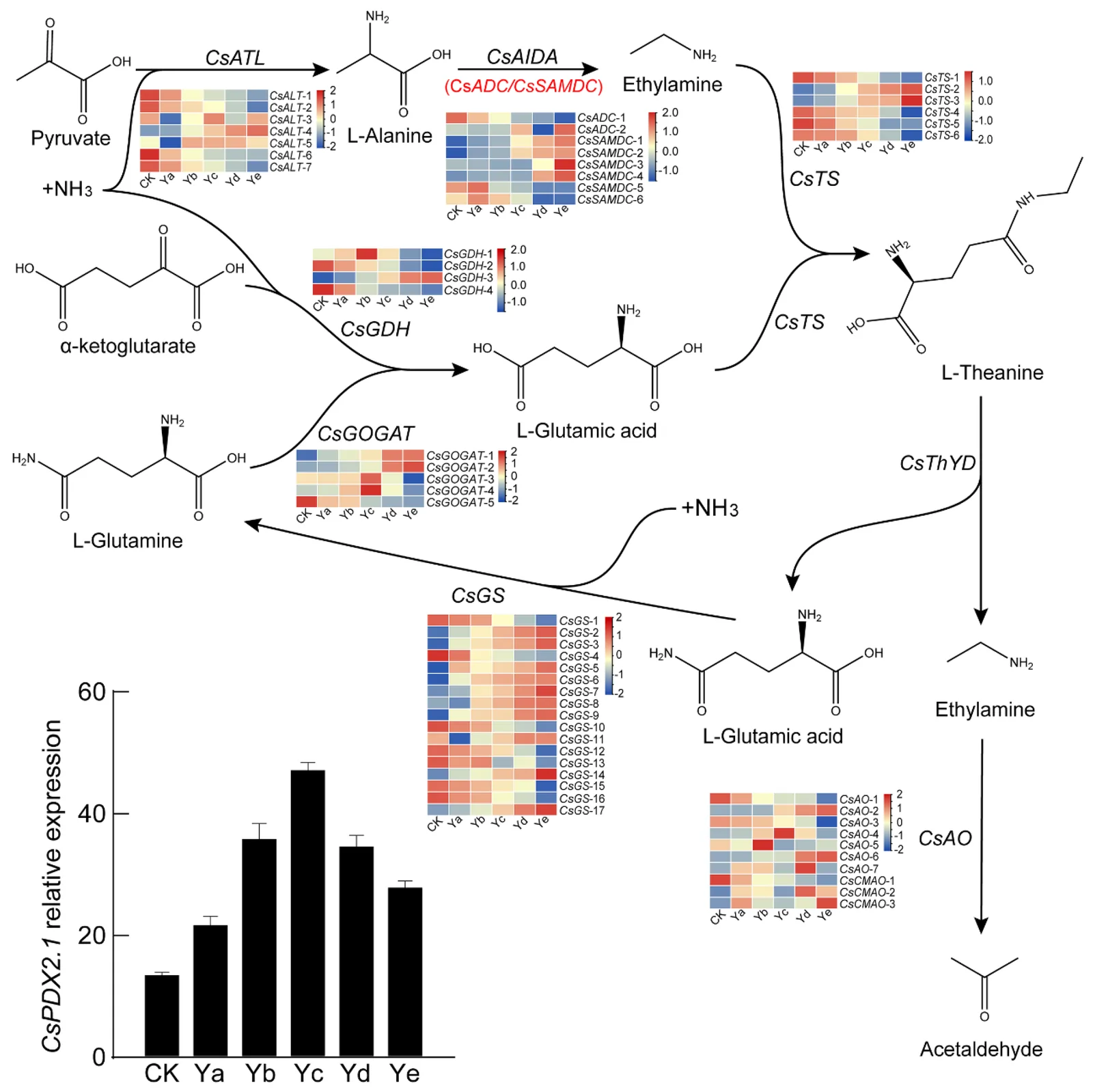
Expression patterns of genes involved in L-theanine biosynthesis and hydrolysis. *CsTS* and *CsGS* (biosynthesis) increased toward Yd (8 h), while *CsPDX2.1* and *CsAO* (hydrolysis) peaked at Yc (6 h) and declined thereafter. Red: high expression; blue: low expression. CK, fresh leaves (0 h); Ya–Ye, 2–10 h withering.

**Figure 7:**
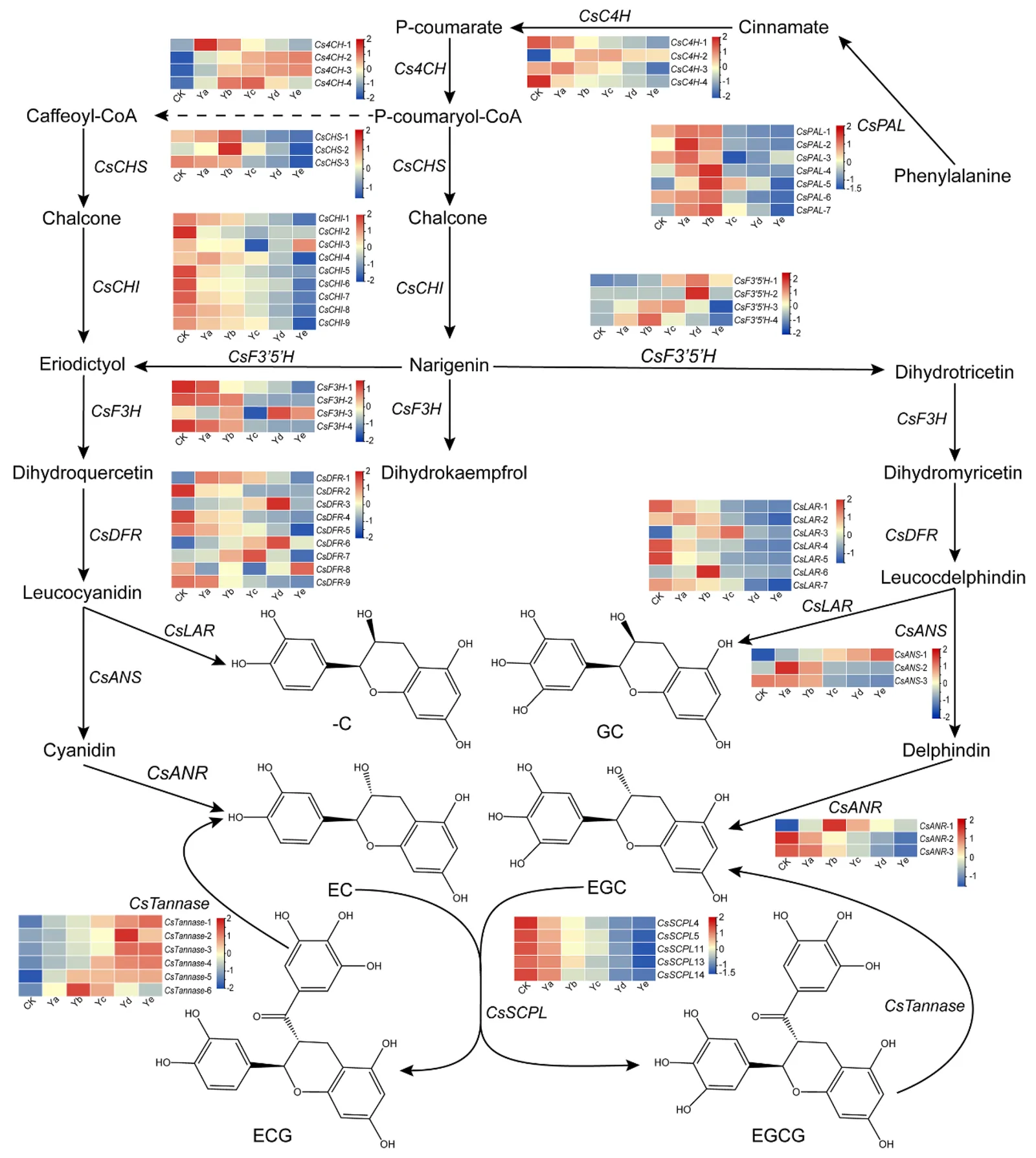
Catechin biosynthetic pathway and related gene expression patterns. *CsTA* (tannase) hydrolyzes galloylated catechins (EGCG, ECG) to non-galloylated forms (EGC, EC) and showed increased expression at Yc–Yd; *CsSCPL* catalyzes the reverse reaction and decreased during withering. Red: high expression; blue: low expression. CK, fresh leaves (0 h); Ya–Ye, 2–10 h withering.

Based on the WGCNA results, the royalblue, salmon, skyblue, and darkorange modules were further analyzed. Genes with high module membership (KME) values were prioritized, and regulatory networks were constructed for theanine and catechin metabolism. Unigenes with FPKM > 15 were selected for pathway visualization (Figure 6 and Figure 7).

In the theanine metabolic pathway (Figure 6), *CsPDX2.1* (CSS003722), which encodes a key enzyme involved in L-theanine hydrolysis, showed an initial increase followed by a decrease in expression during withering, peaking at 6 h (Yc). This expression pattern inversely correlated with theanine content, consistent with its previously reported association in theanine degradation during withering [17]. Conversely, genes involved in theanine biosynthesis, including *CsTS*, *CsGS*, *CsGDH*, and *CsGOGAT*, exhibited expression patterns positively correlated with theanine accumulation, with relatively higher expression levels observed at 8 h (Yd) [31]. Additionally, *CsAO* (amine oxidase) transcripts, which encode an enzyme involved in ethylamine oxidation, were highly expressed in Ya, Yb and Yc, potentially contributing to the downstream metabolism of ethylamine released from theanine hydrolysis during early withering stages [34,35].

In the catechin metabolic pathway (Figure 7), genes involved in the conversion between galloylated and non-galloylated catechins were differentially expressed. *CsTA* (Camellia sinensis tannase), which hydrolyzes galloylated catechins (EGCG and ECG) to their non-galloylated forms (EGC and EC), showed increased expression with prolonged withering, particularly in Yc and Yd [15,16]. This upregulation coincided with the observed decrease in galloylated catechin content. In contrast, *CsSCPL*, which catalyzes the galloylation of non-galloylated catechins, exhibited decreased expression during withering, thereby limiting the re-esterification of less bitter catechins [16]. Structural genes in the flavonoid biosynthesis pathway, including *CsPAL*, *CsC4H*, *CsCHS*, *CsCHI* and *CsF3H*, generally decreased in expression with prolonged withering, whereas *CsLAR*, *CsANS*, and *CsANR*, which are unique to catechin biosynthesis, also showed declining trends [36,37].

The integrated transcriptomic and metabolomic analysis suggested a molecular roadmap underlying the flavor transition during NNT withering. The down regulation of *CsPDX2.1* and *CsAO* after 6 h was associated with reduced theanine hydrolysis, whereas the sustained expression of biosynthetic genes such as *CsTS* and *CsGS* correlated with its accumulation, contributing to the sweet and umami taste by 8 h. Concurrently, the upregulation of *CsTA* was correlated with the hydrolysis of bitter-tasting galloylated catechins, while the suppressed expression of *CsSCPL* was associated with reduced reformation, collectively diminishing bitterness. Thus, the optimal flavor quality at 8 h of withering appears to be associated with the finely tuned temporal expression of these critical genes, which may influence substrate availability and catalytic direction in the metabolic network, potentially guiding the biochemical cascade toward the formation of sweet and mellow sensory attributes.

### 3.6. qRT-PCR Analysis of Genes Related to Differential Metabolite Synthesis

**Figure 8:**
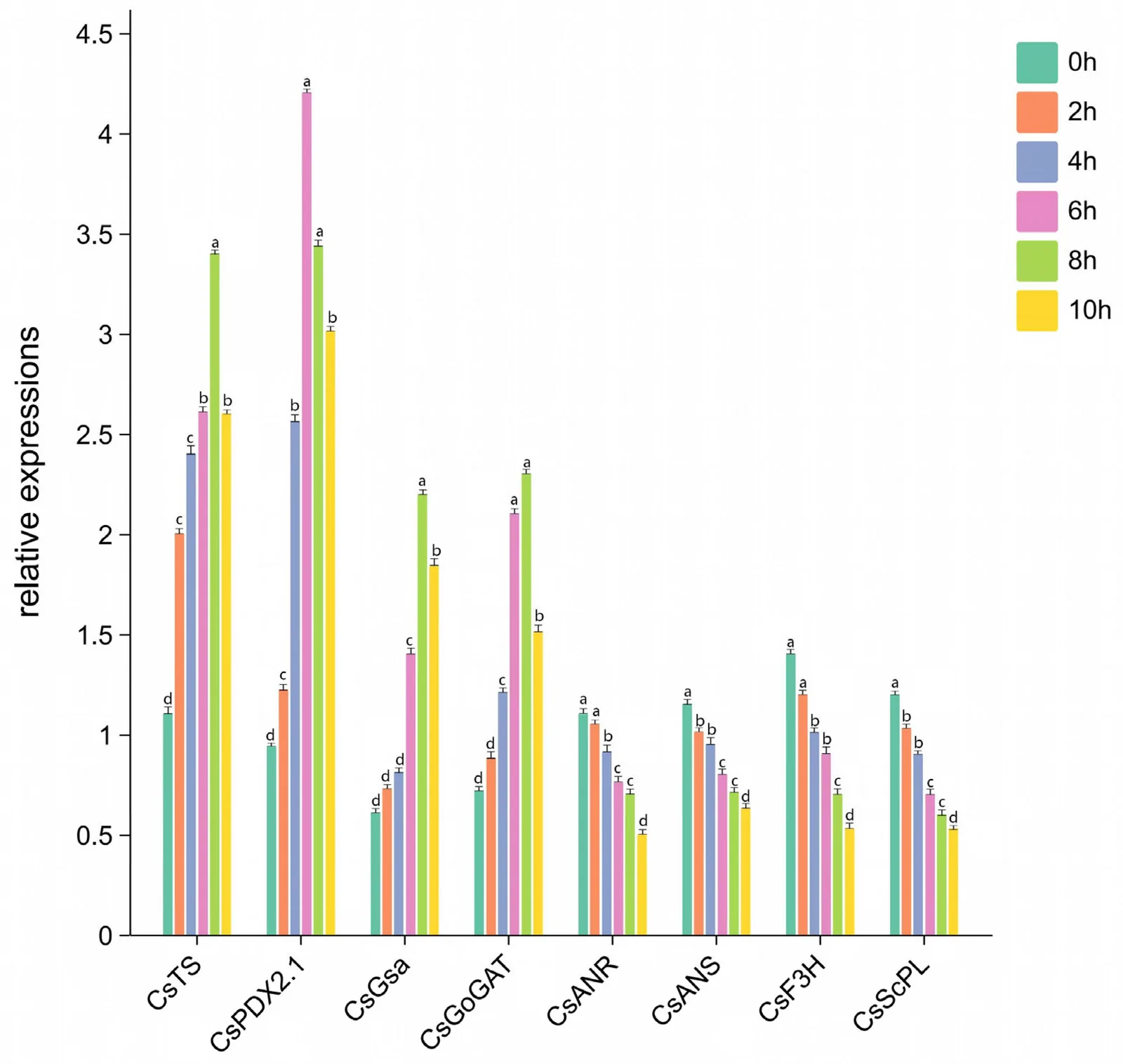
Validation of differentially expressed genes by quantitative real-time PCR (qRT-PCR). The bar charts represent relative expression levels determined by qRT-PCR. Data are presented as mean ± standard deviation (SD) of three biological replicates. *CsTS*, theanine synthase; *CsPDX2.1,* theanine hydrolase; *CsGOGAT*, glutamate synthase; *CsGs*, glutamine synthetase; *CsANR*, anthocyanidin reductase; *CsANS*, anthocyanidin synthase; *CsF3H,* flavanone 3-hydroxylase; *CsSCPL*, serine carboxypeptidase-like acyltransferase. The high consistency between qRT-PCR and RNA-seq data (Pearson’s r = 0.91, *p* < 0.01, n = 48 paired data points) confirms the reliability of the transcriptomic analysis. CK, fresh leaves (0 h); Ya–Ye, 2–10 h withering. Different lowercase letters indicate statistically significant differences (*p* < 0.05).

To validate the reliability of the transcriptome data, eight differentially expressed genes (DEGs) involved in the biosynthesis of key taste related metabolites were selected for quantitative real-time PCR (qRT-PCR) analysis. These included *CsTS* (theanine synthase), *CsPDX2.1* (theanine hydrolase), *CsGOGAT* (glutamate synthase), *CsGs* (glutamine synthetase), *CsANR* (anthocyanidin reductase), *CsANS* (anthocyanidin synthase), *CsF3H* (flavanone 3-hydroxylase), and *CsSCPL* (serine carboxypeptidase-like acyltransferase). The expression dynamics of these genes were analyzed across all withering time points, and the qRT-PCR results were compared with the corresponding RNA-seq data (Figure 8).

As shown in Figure 8, the qRT-PCR results exhibited high consistency with the transcriptomic profiles, with both methods revealing similar temporal expression patterns. Specifically, *CsTS* expression increased progressively during withering, peaking at 8 h (Yd), which aligned with the peak accumulation of theanine observed in metabolomic analysis. Conversely, *CsPDX2.1*, involved in theanine hydrolysis, showed elevated expression during early withering (2–6 h), followed by a decline after 6 h, consistent with the observed reduction in theanine degradation and subsequent theanine accumulation. *CsGOGAT* and *CsGs*, both involved in glutamate and glutamine metabolism, exhibited increasing expression from 6 h onward, supporting the enhanced biosynthesis of amino acids during the later withering stages.

In the catechin metabolic pathway, *CsANR* and *CsANS*, which are involved in the final steps of catechin biosynthesis, showed peak expression at 2–4 h followed by a progressive decline, mirroring the initial increase and subsequent decrease in non-galloylated catechin content. *CsF3H*, an early structural gene in flavonoid biosynthesis, exhibited a gradual decrease in expression with prolonged withering, consistent with the overall reduction in catechin biosynthesis. *CsSCPL*, which catalyzes the galloylation of catechins, showed decreased expression during withering, correlating with the observed reduction in galloylated catechin content. The strong correlation between qRT-PCR and RNA-seq data across all tested genes (Pearson’s r = 0.91, *p* < 0.01, n = 8 genes × 6 time points) confirms the reliability of the transcriptomic dataset and supports the robustness of the gene expression patterns discussed in Section 3.5. These findings further support the key regulatory roles of these genes in modulating the sweet-mellow flavor formation during NNT withering.

### 3.7. Discussion

Our integrated multi-omics analysis suggested that withering duration may critically influence the sweet-mellow taste quality of NNT by coordinately modulating amino acid and catechin metabolism. The sensory-optimized 8 h time point coincided with peak accumulation of theanine, L-aspartic acid, and L-arginine, which are known contributors to sweet and umami taste in tea infusions [3,29,30]. Concurrently, galloylated catechins, the primary sources of bitterness and astringency, declined progressively, with their conversion associated with upregulation of *CsTA* and downregulation of *CsSCPL*. This regulatory pattern aligns with previous reports in other tea varieties [15,16], but our data further suggest that this coordination is temporally fine-tuned during withering, with *CsPDX2.1* downregulation after 6 h may serve as a key switch that shifts theanine flux from hydrolysis toward accumulation.

The temporal peak of pathway enrichment at 8 h may reflect a critical physiological window during withering, when the leaf has undergone sufficient dehydration, potentially activating full stress-responsive metabolism but has not yet entered the degradative phase observed at 10 h. At this time point, the co-enrichment of amino acid biosynthesis and ABC transporter pathways may ensure both the synthesis and intracellular redistribution of sweet-tasting amino acids, while the sustained activity of flavonoid biosynthesis may promote the enzymatic conversion of astringent catechins. This coordinated metabolic state appears to enhance sweetness perception through two mechanisms: (i) the accumulation of theanine, L-aspartic acid, and L-arginine is known to contribute to the sweet and umami taste profiles of tea infusions [3,29,30]; and (ii) the concurrent reduction in galloylated catechins is associated with diminished bitterness and astringency [3,13]. The decline at 10 h, marked by catabolic pathway enrichment, may explain why excessive withering compromises flavor quality.

Notably, the optimal withering duration for NNT (8 h) is longer than typical durations reported for small-leaf yellow teas such as Mengding Huangya and Huoshan Huangya [3,4]. This difference likely reflects the distinct physiological traits of large-leaf assamica materials, including thicker leaf cuticles, higher initial moisture content, and more robust secondary metabolite pools [20], which may require extended dehydration to activate sufficient enzymatic activity while avoiding excessive metabolite degradation. These findings underscore the importance of variety-specific processing parameters rather than applying a one-size-fits-all approach across yellow tea types.

We acknowledge that this study was conducted using a single tea population (*Camelia sinensis* var. assamica) from one location (Poliu Village, Zhenfeng, Guizhou) and a single harvest season (July), which may limit the generalizability of our conclusions. Seasonal variations in temperature and humidity could affect moisture loss and enzymatic activity during withering, potentially shifting the optimal duration. Growing location, including altitude, soil composition, and microclimate, also shapes the baseline metabolic profiles of leaves and may alter their responsiveness to processing [20]. Additionally, genetic diversity among different assamica populations or cultivars may lead to differential expression of key regulatory genes (*CsTA*, *CsSCPL*, *CsPDX2.1*), resulting in population-specific optima. Therefore, while our 8 h optimum provides a robust reference for the studied NNT population, future research across multiple seasons, locations, and assamica germplasms is essential to validate and refine these parameters for broader application. Functional validation via gene silencing or overexpression would also be necessary to establish causal relationships between the identified candidate genes and flavor formation. More broadly, our metabolomic data represent steady-state concentrations rather than metabolic fluxes, which constrains the interpretation of precursor-product relationships such as those inferred between theanine hydrolysis, glutamate accumulation, and subsequent amino acid biosynthesis. While the observed temporal correlations are consistent with the known roles of glutamate as a central hub in theanine and amino acid metabolism, isotope tracing (e.g., ^13^C-glutamate or ^15^N-theanine) and flux analysis would be required for definitive confirmation of these metabolic conversions. Nonetheless, the strong concordance between gene expression dynamics and metabolite profiles provides a robust foundation for generating testable hypotheses regarding the regulatory mechanisms that underpin flavor formation during withering.

## 4. Conclusions

This study integrated metabolomics, transcriptomics, and sensory evaluation to elucidate the molecular basis of sweet-mellow taste formation during withering of Niangniang tea (NNT). Our findings demonstrate that withering for 6–8 h, with 8 h yielding the highest sensory scores, significantly enhances the sweet-mellow flavor through the synergistic accumulation of theanine, L-aspartic acid, and L-arginine, alongside a progressive reduction in bitter-tasting galloylated catechins. Transcriptomic analysis revealed a coordinated regulatory network potentially associated with these metabolic shifts. *CsTS* and *CsGS* are associated with theanine biosynthesis, while *CsPDX2.1* and *CsAO* may be involved in its hydrolysis. In catechin metabolism, *CsTA* is associated with the conversion of galloylated catechins to less bitter non-galloylated forms, whereas *CsSCPL* may be involved in their re-esterification. The temporal coordination of these genes, particularly the downregulation of *CsPDX2.1* after 6 h and upregulation of *CsTA* toward 8 h, may contribute to the balance between sweetness and astringency, potentially shaping the final flavor profile. The 8 h optimum identified for this large-leaf assamica variety exceeds the typical durations for small-leaf yellow teas, reflecting the distinct physiological properties of large-leaf materials and underscoring the necessity of variety-specific processing optimization. Our work establishes a data-driven framework for withering optimization in NNT production and demonstrates the power of multi-omics integration for precision processing in specialty teas. Future research across multiple seasons, locations, and assamica germplasms will be essential to validate and refine these parameters for broader application.

## Data Availability

The original contributions presented in this study are included in the article/Supplementary Material. Further inquiries can be directed to the corresponding authors.

## Supplementary Materials

The following supporting information can be downloaded at: https://www.mdpi.com/article/10.3390/foods15162896/s1, Table S1. Cationic differential metabolites identified by metabolomics of Niangniang tea. Supplementary Table S2. Differentially expressed genes identified by transcriptome of Niangniang tea.
